# Unravelling HP1 functions: post-transcriptional regulation of stem cell fate

**DOI:** 10.1007/s00412-021-00760-1

**Published:** 2021-06-15

**Authors:** Assunta Maria Casale, Ugo Cappucci, Lucia Piacentini

**Affiliations:** Department of Biology and Biotechnology “C. Darwin”, Sapienza University of Rome, Rome, Italy

**Keywords:** Heterochromatin protein 1, Heterochromatin, Post-transcriptional regulation of gene expression, Germline stem cells, *Drosophila melanogaster*

## Abstract

Heterochromatin protein 1 (HP1) is a non-histone chromosomal protein first identified in *Drosophila* as a major component of constitutive heterochromatin, required for stable epigenetic gene silencing in many species including humans. Over the years, several studies have highlighted additional roles of HP1 in different cellular processes including telomere maintenance, DNA replication and repair, chromosome segregation and, surprisingly, positive regulation of gene expression. In this review, we briefly summarize past research and recent results supporting the unexpected and emerging role of HP1 in activating gene expression. In particular, we discuss the role of HP1 in post-transcriptional regulation of mRNA processing because it has proved decisive in the control of germline stem cells homeostasis in *Drosophila* and has certainly added a new dimension to our understanding on HP1 targeting and functions in epigenetic regulation of stem cell behaviour.

## Introduction

Heterochromatin protein 1 (also known as HP1a), encoded by the *Su(var)2–5* gene, is an evolutionarily conserved chromosomal protein first identified in *Drosophila melanogaster* by its association with constitutive heterochromatin domains and through mutations acting as dosage-dependent modifiers of position-effect variegation (James and Elgin 1986; James et al. 1989; Eissenberg et al. 1990). Numerous studies have shown that such protein is highly conserved (Singh et al. 1991; Eissenberg and Elgin 2000; Wang et al. 2000); orthologues of HP1 were discovered in *Schizosaccharomyces pombe* (Swi6) (Lorentz et al. 1994), *Xenopus* (Xhp1α and Xhp1γ) (Meehan et al. 2003), Chicken (CHCB1, CHCB2 and CHCB3) (Yamaguchi et al. 1998) and *Tetrahymena* (Pdd1p) (Huang et al. 1999), with the exception of budding yeast, *Saccharomyces cerevisiae*, in which the organization of silenced chromatin domains depends on SIR proteins (see (Kueng et al. 2013) for a review). In mammals, there are three paralogues, HP1α, HP1β and HP1γ, encoded by the *CBX5*, *CBX1* and *CBX3* genes, respectively (Singh et al. 1991; Saunders et al. 1993; Ye and Worman 1996; Li et al. 2002; Maison and Almouzni 2004).

HP1 proteins are mainly involved in heterochromatin structural organization and epigenetic gene silencing (Wang et al. 2000; Bannister et al. 2001; Lachner et al. 2001; Lomberk et al. 2006). According to the general model proposed for heterochromatin formation, histone methyltransferases (HMTases) methylate the histone H3 at lysine 9 (H3K9me2/3), creating selective binding sites for themselves and for the HP1 chromodomain (see (Jenuwein 2001)for a review). The HP1-H3K9me2/3 complex serves as a binding platform for the recruitment of other heterochromatic factors and represents the early step in the cascade of molecular events, leading to the establishment of heterochromatin domains and epigenetic repression of transcriptional activity (Nakayama et al. 2001; Czermin et al. 2001; Hall et al. 2002; Snowden et al. 2002; Schotta et al. 2003; Stewart et al. 2005; Fanti and Pimpinelli 2008; Motamedi et al. 2008). Recent studies in different model organisms including *Schizosaccharomyces pombe*, *Drosophila* and mammals have proposed a new mechanism for heterochromatin compartmentalization and spreading based on the ability of higher-order HP1 oligomers to aggregate into liquid-phase droplets that contribute to heterochromatin phase separation and promote epigenetic gene silencing (Larson et al. 2017; Strom et al. 2017; Tatarakis et al. 2017; Sanulli et al. 2019). All these data, consistent with HP1 tethering experiments that led to transcriptional silencing of reporter genes (Lehming et al. 1998; Seeler et al. 1998; van der Vlag et al. 2000; Li et al. 2003; Danzer and Wallrath 2004), support a model whereby HP1 proteins primarily act as transcriptional repressor in nucleation and spreading of silent chromatin.

Notably, in *Schizosaccharomyces pombe*, the HP1 orthologue Swi6 induces the epigenetic silencing of heterochromatin domains not only at transcriptional level (Bühler et al. 2006) but also at post-transcriptional level (Keller et al. 2012). In fact, Keller and collaborators have demonstrated that Swi6, through its hinge domain, is capable of capturing transcripts from heterochromatic genes and directing them to the nuclear exosome for degradation (Keller et al. 2012).

In addition to being required for heterochromatin formation and epigenetic gene silencing, *Drosophila* HP1 plays an essential role in both telomere capping and telomere elongation (Fanti et al. 1998; Savitsky et al. 2002; Perrini et al. 2004; Canudas et al. 2011; Chow et al. 2018). Based on the model proposed by Perrini et al. (Perrini et al. 2004), *Drosophila* HP1 controls telomere capping and transcriptional silencing of telomeric retroelements by two different mechanisms: telomere capping results from the direct binding of HP1 hinge domain to telomeric single-strand DNA sequences, while epigenetic silencing of telomeric retrotransposons essentially depends on the dynamic interaction of the HP1 chromodomain with H3K9me3 nucleosomes in telomeric heterochromatin (Perrini et al. 2004).

Although initially identified in the context of the heterochromatin-dependent gene silencing and in addition to its role in telomere integrity maintenance, it is now evident that HP1 protein has additional nuclear functions including DNA replication and repair (Schwaiger et al. 2010; Dronamraju and Mason 2011; Pokholkova et al. 2015; Bosso et al. 2019), chromosome segregation (Kellum and Alberts 1995; Kellum et al. 1995), transcriptional activation and elongation (Lu et al. 2000; Piacentini et al. 2003; Cryderman et al. 2005; De Lucia et al. 2005; Johansson et al. 2007; de Wit et al. 2007; Lin et al. 2008, 2012; Piacentini and Pimpinelli 2010; Kwon et al. 2010) and RNA stability (Piacentini et al. 2009; Casale et al. 2019).

The functional versatility of HP1 arises mainly from its structural plasticity; HP1 possesses, in fact, a characteristic modular architecture consisting of two functional domains: an amino-terminal chromodomain (CD), important for the binding to the N-terminal tail of histone H3 when it is di- or trimethylated (Bannister et al. 2001; Lachner et al. 2001; Jacobs et al. 2001; Nielsen et al. 2002; Jacobs and Khorasanizadeh 2002), and a C-terminal globular chromo shadow domain (CSD) (Aasland and Stewart 1995) which contains a PxVxL degenerate hydrophobic pentapeptide motif necessary for HP1 dimerization and protein–protein interactions (Smothers and Henikoff 2000; Cowieson et al. 2000). Chromo- and chromoshadow domains are interconnected by a short and less conserved hinge region that confers to HP1, the necessary structural flexibility to adapt itself to specific chromatin contexts through the interaction with different protein partners (Smothers and Henikoff 2001; Nishibuchi and Nakayama 2014). In addition to protein–protein interactions, several reports in *Drosophila*, *Schizosaccharomyces pombe* and mammals have revealed that HP1 proteins exhibit nucleic acid binding activity, most often involving the hinge region but sometimes either the CD or CSD domains. For instance, in *Drosophila*, in vivo and in vitro studies have demonstrated that HP1 directly binds nascent RNAs through its CD and telomeric DNA sequences via its hinge domain (Piacentini et al. 2003, 2009; Perrini et al. 2004; Casale et al. 2019). Other than with nascent transcripts from protein-coding genes, HP1 has been found selectively associated with a broad set of RNAs transcribed from repetitive regions (Alekseyenko et al. 2014).

As *Drosophila* HP1, also HP1 orthologues in other species display nucleic acid binding activity. In *Saccharomyces pombe*, Swi6 is able to bind RNAs through its hinge region (Keller et al. 2012; Kumar et al. 2020), and, in mammals, HP1α, SUMOylated in the hinge domain, targets pericentromeric heterochromatin by interacting with long nuclear noncoding transcripts corresponding to major satellite repeats (Maison et al. 2002, 2011; Muchardt et al. 2002). Moreover, the unstructured hinge domain, necessary for the targeting of HP1α to constitutive heterochromatin, is also required for the interaction with parallel G-quadruplex structures formed by the TElomericRepeat-containing RNA (TERRA) transcribed from telomeres (Roach et al. 2020).

A further level of HP1 functional complexity is achieved through multiple covalent post-translational modifications (PTMs) that are very important in modulating both HP1 interactions and chromatin binding ability (see (Sales-Gil and Vagnarelli 2020) for a review). The phosphorylation, predominantly at serine and threonine residues in the hinge domain, is the most abundant and well-studied HP1 post-translational modifications; it has been described for the first time in the mid-1990s for its functional importance in heterochromatin formation in *Drosophila* embryos (Eissenberg et al. 1994). It was also found that differentially phosphorylated HP1 isoforms affect HP1 protein interactions and chromosomal distribution, other than HP1 silencing activity (Zhao and Eissenberg 1999; Zhao et al. 2001; Badugu et al. 2005). In *Saccharomyces pombe*, Swi6 phosphorylation specifically controls transcriptional gene silencing in heterochromatin (Shimada et al. 2009) and provides a dynamic pathway for the differential regulation of heterochromatin in response to inter- and intracellular signals (Shimada and Murakami 2010).

Also mammalian HP1 isoforms undergo specific modifications including phosphorylation, acetylation, methylation, formylation, ubiquitination, SUMOylation and citrullination (Minc et al. 1999; Lomberk et al. 2006; LeRoy et al. 2009; Maison et al. 2011; Wiese et al. 2019; Sales-Gil and Vagnarelli 2020). Similarly to *Drosophila* HP1, each of these modifications can change HP1 functions, thus creating an epigenetic subcode that would permit different interactions of HP1 in different chromatin contexts. For example, specific phosphorylation of four amino acid residues in the N-terminal tail of HP1α are crucial for its binding to H3K9me3 and heterochromatin formation (Minc et al. 1999; Li et al. 2002; Hiragami-Hamada et al. 2011; Nishibuchi et al. 2014; Bryan et al. 2017). Moreover, the phosphorylation of HP1α within its hinge domain is required for proper localization to centromeres during mitosis in mammalian cells (Chakraborty et al. 2014), while the phosphorylation of HP1γ regulates transcription of distinct gene subsets during differentiation programs (Seo et al. 2018).

## The other side of HP1 functions: the positive regulation of gene expression

In contrast to the most commonly cited role in heterochromatin formation and gene silencing, a growing body of evidence in flies, mammals and other organisms has highlighted the importance of HP1 proteins in promoting gene expression. For instance, in *Drosophila*, it has been shown that mutations in HP1-encoding gene cause a significant downregulation of heterochromatic genes such as *light* and *rolled*, supporting the idea that some genes depend on their heterochromatic context for efficient expression (Hearn et al. 1991; Clegg et al. 1998; Lu et al. 2000). The role of HP1 in promoting expression of heterochromatic sequences has been reported also for HP1 paralogues and orthologues. Rhino (also known as HP1d), a female germline-specific paralogue of *Drosophila* HP1, mediates Pol II-dependent transcription of the dual-strand piRNA clusters by recruiting Moonshiner and TBP-related factor 2 (TRF2) to heterochromatin (Klattenhoff et al. 2009; Andersen et al. 2017). Likewise, fission yeast Swi6 promotes Pol II-mediated transcription of heterochromatic inverted repeats by recruiting the anti-silencing factor Epe1 that associates with SAGA to regulate transcription within heterochromatin and to restrain the spread of pericentromeric heterochromatin boundary (Zofall and Grewal 2006; Isaac et al. 2007; Trewick et al. 2008; Bao et al. 2019). All together, these results suggest that HP1 proteins may function as positive transcriptional regulators of heterochromatic sequences.

Furthermore, accumulating evidence in *Drosophila* suggests that HP1 plays a direct role in the maintenance of active transcription of several euchromatic genes involved in chromatin dynamics and cell-cycle progression (Cryderman et al. 2005; De Lucia et al. 2005). Consistent with these results, Liu et al. have highlighted for HP1 also a sex-specific role in regulating chromatin structure and gene transcription (Liu et al. 2005).

The role of HP1 in gene expression is complex and not completely understood but seems to involve at least two different mechanisms.

As a transcriptional activator, HP1 might directly mediate the recruitment of transcriptional factors or co-activators to specific regulatory regions of a gene, thus promoting active transcription (Kwon et al. 2010; Ilyin et al. 2020; Schoelz et al. 2020). For instance, Kwon et al. revealed that in *Drosophila*, all HP1 paralogues (HP1a, HP1b and HP1c) control the stable recruitment of the histone chaperone complex FACT (facilitates chromatin transcription) on active chromatin, thus promoting gene expression (Kwon et al. 2010). Also in planaria, through the functional association with the FACT complex, HP1 triggers regenerative proliferation of adult stem cells activating *Mcm5* expression during transcription elongation (Zeng et al. 2013). In addition, it has been demonstrated that *Drosophila* HP1 co-localizes with stalled polII on chromatin immediately downstream of TSSs, implicating a regulatory function of HP1 in controlling RNA polII elongation (Yin et al. 2011).

Alternatively, HP1 might work at a post-transcriptional level regulating folding, modification, processing and stability of newly synthesized RNAs. The first compelling evidence of an involvement of HP1 in chromatin-associated post-transcriptional regulation of gene expression was provided in *Drosophila* by Piacentini et al. (Piacentini et al. 2003, 2009; Piacentini and Pimpinelli 2010) who have found a novel mechanism for HP1-mediated gene expression. They found HP1 specifically associated with induced, actively transcribed genes, including transgenic, developmental and heat-shock-induced puffs on polytene chromosomes from the third instar larvae salivary glands (Fanti et al. 2003; Piacentini et al. 2003). Intriguingly, they demonstrated that HP1 is co-transcriptionally recruited on nascent transcripts and identified in the chromodomain the module of HP1 directly involved in RNA binding in vivo, since RNase treatment or chromodomain mutations completely abolished HP1 recruitment on active chromatin (Piacentini et al. 2003). Moreover, they identified more than one hundred HP1 target genes whose transcripts are co-transcriptionally stabilized by an heterogeneous nuclear ribonucleoprotein (hnRNP) complex containing HP1 together with DDP1 (Cortes et al. 1999), HRB87F (Haynes et al. 1991) and PEP (Amero et al. 1991), which belong to different classes of hnRNPs known to be involved in RNA packaging, stability and processing (Piacentini et al. 2009; Piacentini and Pimpinelli 2010).

## Epigenetic regulation of germline stem cell maintenance: a new dimension which broadens our understanding of HP1 functional versatility

**Fig. 1:**
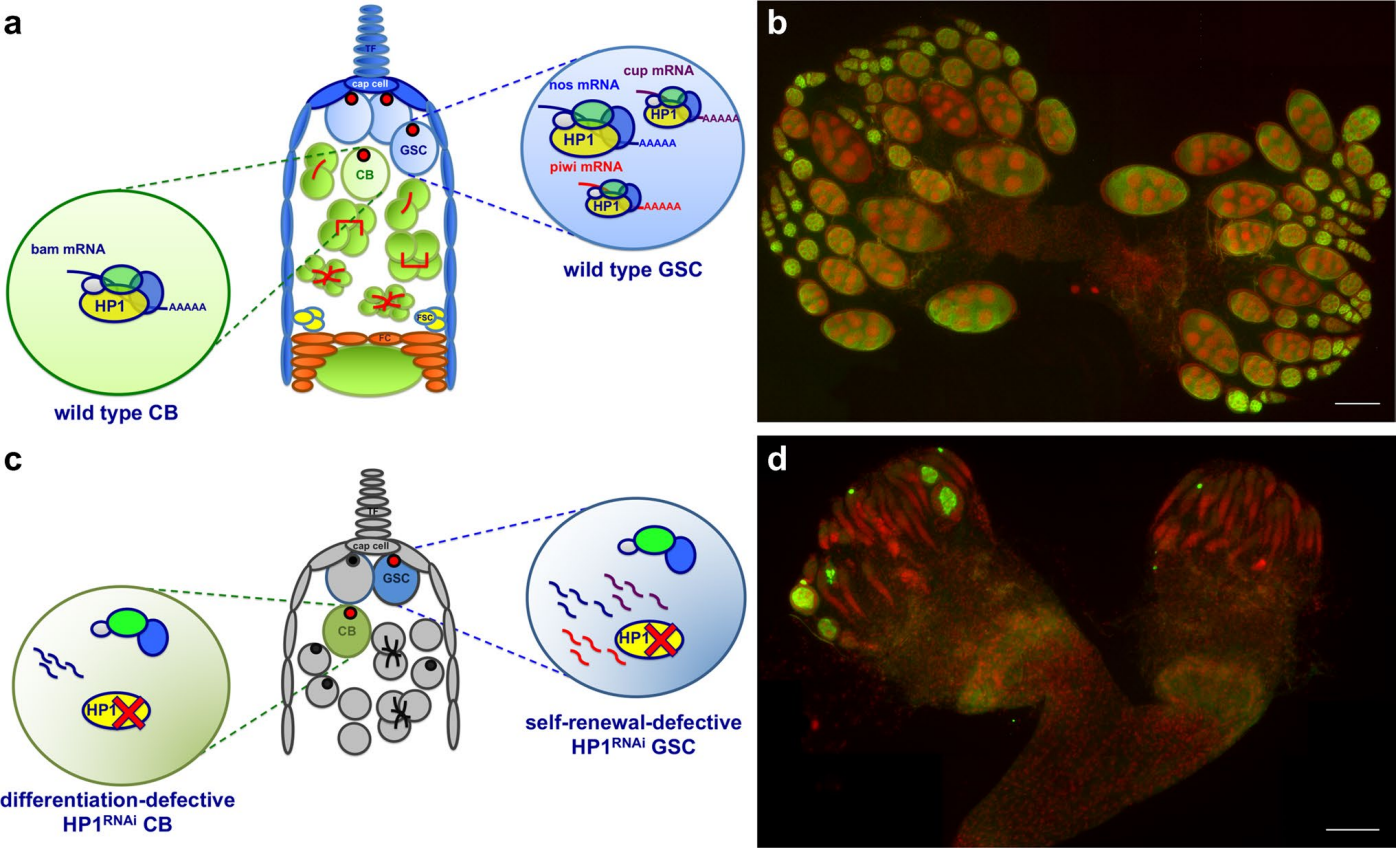
HP1 is required for correct ovarian development in *Drosophila*. **a** Schematic representation of the HP1-dependent post-transcriptional regulation of germline stem cells (GSCs); in wild-type condition, HP1 binds and stabilizes the transcripts of key genes regulating the balance between GSC self-renewal (*nos*, *piwi* and *cup*) and differentiation (*bam*). **b** Developing wild-type ovaries obtained from 72–96-h old pupae stained for the germ cell marker Vasa (green) and DNA (red). **c** HP1 functional inactivation induces premature RNA degradation leading to a failure in the self-renewal/differentiation switch program. **d** HP1 depleted ovaries stained for Vasa (green) and DNA (red). As compared to the control (**b**), the majority of the ovarioles are completely devoid of germ cells. Scale bars, 100 μm. GSC, germ stem cell; CB, cystoblast; TF, terminal filament cells

The emerging role of HP1 in regulating co-transcriptionally RNA packaging and stability has also proved decisive in the control of female germline stem cells homeostasis in *Drosophila* (Casale et al. 2019). The stem cell’s behaviour is a highly dynamic process, implying intricate networks of extrinsic signalling, transcriptional, post-transcriptional and translational regulations (see (Blatt et al. 2020) for a review). In *Drosophila* germline stem cells (GSCs), multiple layers of post-transcriptional regulation, including alternative splicing, RNA modifications and translational repression, orchestrate the balance between self-renewal and differentiation and ensure proper germline stem cell homeostasis (Blatt et al. 2020). Notably, messenger RNA stability is a very important control point in modulating gene expression in GSCs, and, in this context, HP1 has emerged as a key regulator (Casale et al. 2019). In fact, it has been shown that HP1 is intrinsically required for chromatin-associated post-transcriptional regulation of female germline stem cell maintenance in *Drosophila* (Casale et al. 2019). Unexpectedly, it has been demonstrated that HP1 exerts this pivotal function by positively regulating the packaging and stability of newly synthesized transcripts involved in GSC self-renewal and differentiation such as *cup*, *nanos (nos)*, *piwi* and *bag of marbles* (*bam)* (Fig. 1). Consistent with the above mentioned findings, Casale et al. (Casale et al. 2019) confirmed the capacity of HP1 to directly bind the nascent transcripts and provided an important contribution to the understanding of the fundamental mechanisms which control the identity and maintenance of germline stem cells in *Drosophila*. As well as HP1, other genes important for heterochromatin formation and epigenetic gene silencing have been implicated in stem cell maintenance. For instance, it has previously been demonstrated that a H3K9-specific methyltransferase SetB1 and its *Drosophila* homologue Eggless (Egg or dSETDB1) are important for maintaining self-renewal of embryonic stem cells in mice and adult germline stem cells in *Drosophila*, respectively (Bilodeau et al. 2009; Wang et al. 2011). In addition, it has been shown that the DNA-associated protein Stonewall (Stwl), essential for heterochromatin organization in *Drosophila*, is required cell autonomously for GSC maintenance by repressing differentiation genes (Maines et al. 2007) and that constitutive DNA methylation, another epigenetic repressive mark associated with heterochromatin formation, is essential for self-renewal of mouse haematopoietic stem cell (Bröske et al. 2009). These findings suggest that epigenetic gene repression mechanisms, often associated with heterochromatin formation, might be a conserved mechanism for stem cell self-renewal. In all these cases, however, the role of heterochromatic genes in promoting self-renewal is mainly based on the epigenetic repression of differentiation genes. The results of Casale et al. (Casale et al. 2019), on the contrary, have added a new dimension to our understanding of HP1 targeting and functions because, for the first time, they highlighted a novel and unexpected role of HP1 in chromatin-associated post-transcriptional regulation of key genes controlling the balance of self-renewal and differentiation in *Drosophila* germline stem cells.

## Conclusions

Since its identification, during 1980s, the multifunctionality of HP1 is still a subject of new discoveries. The ability of HP1 to maintain a silenced state or promote rapid transcription upon cell insult or cell fate program is a very fascinating field. What molecular mechanisms are responsible for the functional versatility of this protein? The details of how HP1 regulates active transcription remain largely unknown. Post-translational modifications certainly play a key role in modulating HP1 functions because they can differentially regulate HP1 activity, localization and chromatin interactions. Similarly, the identification of HP1 binding partners would help to provide some explanation on how it works in different chromatin contexts and cellular processes.

**Fig. 2:**
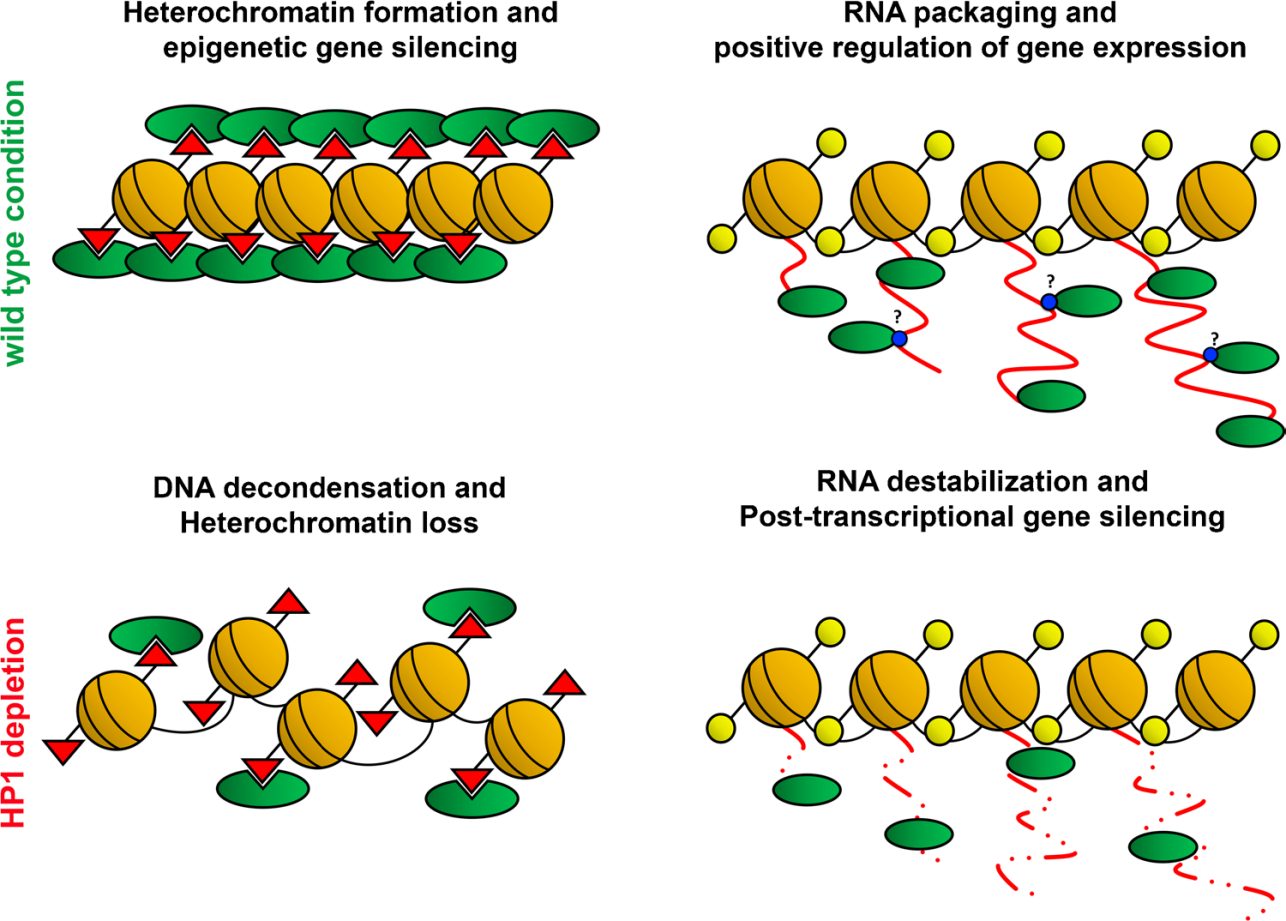
Schematic representation of a tentative model that offers an explanation for the dual role of HP1 in epigenetic gene silencing and positive regulation of gene expression. In heterochromatin domains, HP1 binds to trimethylated H3K9 (red triangles), thus promoting DNA packaging and epigenetic gene silencing. In euchromatin regions, instead, HP1 protects nascent transcripts from premature degradation, thus reinforcing gene expression. According to our model HP1 could hypothetically bind target transcripts by specifically recognizing methylated residues (blue circles). HP1 functional inactivation leads to DNA de-condensation in heterochromatin and post-transcriptional gene silencing in euchromatin. Yellow lollipops depict acetylated histone tails

An interesting aspect to discuss is whether HP1 performs different functions in different chromatin contexts or whether it performs the same function, the nucleic acid packaging, in both euchromatin and heterochromatin. Consistently with this second hypothesis, the DNA compaction in heterochromatin domains could result in large-scale chromatin condensation and epigenetic gene silencing, whilst, in the euchromatin, the pre-mRNA packaging in HP1-containing ribonucleoprotein particles (RNP), could play a dual role: on one hand, it protects the newly synthesized RNA from degradation; on the other hand, it provides the machinery that enables accurate RNA processing in a temporally and spatially regulated fashion, thus reinforcing gene expression (Fig. 2). It remains to be clarified how the direct targeting of HP1 chromodomain to nascent transcripts occurs. Considering the methyl-binding affinity of HP1 proteins, we certainly cannot exclude the possibility that HP1, as an *epigenetics reader*, might specifically recognize and bind, through its chromodomain, methylated residues on target RNAs, thus directly regulating their metabolism and processing. The methylated residues in RNA sequences could in fact allow HP1 to discriminate between different transcripts and to specifically regulate their metabolism and processing (Fig. 2). In conclusion, the dual role of HP1 in epigenetic gene silencing and positive regulation of gene expression could just be two sides of the same HP1 coin.

## Data Availability

Not applicable.

## References

[CR1] Aasland R, Stewart AF (1995). The chromo shadow domain, a second chromo domain in heterochromatin-binding protein 1, HP1. Nucleic Acids Res.

[CR2] Alekseyenko AA, Gorchakov AA, Zee BM (2014). Heterochromatin-associated interactions of Drosophila HP1a with dADD1, HIPP1, and repetitive RNAs. Genes Dev.

[CR3] Amero SA, Elgin SC, Beyer AL (1991). A unique zinc finger protein is associated preferentially with active ecdysone-responsive loci in Drosophila. Genes Dev.

[CR4] Andersen PR, Tirian L, Vunjak M, Brennecke J (2017). A heterochromatin-dependent transcription machinery drives piRNA expression. Nature.

[CR5] Badugu R, Yoo Y, Singh PB, Kellum R (2005). Mutations in the heterochromatin protein 1 (HP1) hinge domain affect HP1 protein interactions and chromosomal distribution. Chromosoma.

[CR6] Bannister AJ, Zegerman P, Partridge JF (2001). Selective recognition of methylated lysine 9 on histone H3 by the HP1 chromo domain. Nature.

[CR7] Bao K, Shan C-M, Moresco J (2019). Anti-silencing factor Epe1 associates with SAGA to regulate transcription within heterochromatin. Genes Dev.

[CR8] Bilodeau S, Kagey MH, Frampton GM (2009). SetDB1 contributes to repression of genes encoding developmental regulators and maintenance of ES cell state. Genes Dev.

[CR9] Blatt P, Martin ET, Breznak SM, Rangan P (2020) Post-transcriptional gene regulation regulates germline stem cell to oocyte transition during Drosophila oogenesis. Curr Top Dev Biol 140:3–34. 10.1016/bs.ctdb.2019.10.003 10.1016/bs.ctdb.2019.10.003 32591078

[CR10] Bosso G, Cipressa F, Moroni ML (2019). NBS1 interacts with HP1 to ensure genome integrity. Cell Death Dis.

[CR11] Bröske A-M, Vockentanz L, Kharazi S (2009). DNA methylation protects hematopoietic stem cell multipotency from myeloerythroid restriction. Nat Genet.

[CR12] Bryan LC, Weilandt DR, Bachmann AL (2017). Single-molecule kinetic analysis of HP1-chromatin binding reveals a dynamic network of histone modification and DNA interactions. Nucleic Acids Res.

[CR13] Bühler M, Verdel A, Moazed D (2006). Tethering RITS to a nascent transcript initiates RNAi- and heterochromatin-dependent gene silencing. Cell.

[CR14] Canudas S, Houghtaling BR, Bhanot M (2011). A role for heterochromatin protein 1γ at human telomeres. Genes Dev.

[CR15] Casale AM, Cappucci U, Fanti L, Piacentini L (2019). Heterochromatin protein 1 (HP1) is intrinsically required for post-transcriptional regulation of Drosophila germline Stem Cell (GSC) maintenance. Sci Rep.

[CR16] Chakraborty A, Prasanth KV, Prasanth SG (2014). Dynamic phosphorylation of HP1α regulates mitotic progression in human cells. Nat Commun.

[CR17] Chow TT, Shi X, Wei J-H (2018). Local enrichment of HP1alpha at telomeres alters their structure and regulation of telomere protection. Nat Commun.

[CR18] Clegg NJ, Honda BM, Whitehead IP (1998). Suppressors of position-effect variegation in Drosophila melanogaster affect expression of the heterochromatic gene light in the absence of a chromosome rearrangement. Genome.

[CR19] Cortes A, Huertas D, Fanti L (1999). DDP1, a single-stranded nucleic acid-binding protein of Drosophila, associates with pericentric heterochromatin and is functionally homologous to the yeast Scp160p, which is involved in the control of cell ploidy. EMBO J.

[CR20] Cowieson NP, Partridge JF, Allshire RC, McLaughlin PJ (2000). Dimerisation of a chromo shadow domain and distinctions from the chromodomain as revealed by structural analysis. Curr Biol.

[CR21] Cryderman DE, Grade SK, Li Y (2005). Role of Drosophila HP1 in euchromatic gene expression. Dev Dyn.

[CR22] Czermin B, Schotta G, Hülsmann BB (2001). Physical and functional association of SU(VAR)3–9 and HDAC1 in Drosophila. EMBO Rep.

[CR23] Danzer JR, Wallrath LL (2004). Mechanisms of HP1-mediated gene silencing in Drosophila. Development.

[CR24] De Lucia F, Ni JQ, Vaillant C, Sun FL (2005). HP1 modulates the transcription of cell-cycle regulators in Drosophila melanogaster. Nucleic Acids Res.

[CR25] de Wit E, Greil F, van Steensel B (2007). High-resolution mapping reveals links of HP1 with active and inactive chromatin components. PLOS Genet.

[CR26] Dronamraju R, Mason JM (2011). MU2 and HP1a regulate the recognition of double strand breaks in Drosophila melanogaster. PLoS One.

[CR27] Eissenberg JC, Elgin SCR (2000). The HP1 protein family: getting a grip on chromatin. Curr Opin Genet Dev.

[CR28] Eissenberg JC, Ge YW, Hartnett T (1994). Increased phosphorylation of HP1, a heterochromatin-associated protein of Drosophila, is correlated with heterochromatin assembly. J Biol Chem.

[CR29] Eissenberg JC, James TC, Foster-Hartnett DM (1990). Mutation in a heterochromatin-specific chromosomal protein is associated with suppression of position-effect variegation in Drosophila melanogaster. Proc Natl Acad Sci U S A.

[CR30] Fanti L, Pimpinelli S (2008). HP1: a functionally multifaceted protein. Curr Opin Genet Dev.

[CR31] Fanti L, Giovinazzo G, Berloco M, Pimpinelli S (1998). The heterochromatin protein 1 prevents telomere fusions in Drosophila. Mol Cell.

[CR32] Fanti L, Berloco M, Piacentini L, Pimpinelli S (2003). Chromosomal distribution of heterochromatin protein 1 (HP1) in Drosophila: a cytological map of euchromatic HP1 binding sites. Genetica.

[CR33] Hall IM, Shankaranarayana GD, Noma K (2002). Establishment and maintenance of a heterochromatin domain. Science.

[CR34] Haynes SR, Johnson D, Raychaudhuri G, Beyer AL (1991). The Drosophila Hrb87F gene encodes a new member of the A and B hnRNP protein group. Nucleic Acids Res.

[CR35] Hearn MG, Hedrick A, Grigliatti TA, Wakimoto BT (1991). The effect of modifiers of position-effect variegation on the variegation of heterochromatic genes of Drosophila melanogaster. Genetics.

[CR36] Hiragami-Hamada K, Shinmyozu K, Hamada D (2011). N-terminal phosphorylation of HP1α promotes its chromatin binding. Mol Cell Biol.

[CR37] Huang H, Smothers JF, Wiley EA, Allis CD (1999). A nonessential HP1-like protein affects starvation-induced assembly of condensed chromatin and gene expression in macronuclei of Tetrahymena thermophila. Mol Cell Biol.

[CR38] Ilyin AA, Stolyarenko AD, Klenov MS, Shevelyov YY (2020). Various modes of HP1a interactions with the euchromatic chromosome arms in Drosophila ovarian somatic cells. Chromosoma.

[CR39] Isaac S, Walfridsson J, Zohar T (2007). Interaction of Epe1 with the heterochromatin assembly pathway in Schizosaccharomyces pombe. Genetics.

[CR40] Jacobs SA, Khorasanizadeh S (2002). Structure of HP1 chromodomain bound to a lysine 9-methylated histone H3 tail. Science.

[CR41] Jacobs SA, Taverna SD, Zhang Y (2001). Specificity of the HP1 chromo domain for the methylated N-terminus of histone H3. EMBO J.

[CR42] James TC, Elgin SC (1986). Identification of a nonhistone chromosomal protein associated with heterochromatin in Drosophila melanogaster and its gene. Mol Cell Biol.

[CR43] James TC, Eissenberg JC, Craig C (1989). Distribution patterns of HP1, a heterochromatin-associated nonhistone chromosomal protein of Drosophila. Eur J Cell Biol.

[CR44] Jenuwein T (2001). Re-SET-ting heterochromatin by histone methyltransferases. Trends Cell Biol.

[CR45] Johansson AM, Stenberg P, Bernhardsson C, Larsson J (2007). Painting of fourth and chromosome-wide regulation of the 4th chromosome in Drosophila melanogaster. EMBO J.

[CR46] Keller C, Adaixo R, Stunnenberg R (2012). HP1Swi6 mediates the recognition and destruction of heterochromatic RNA transcripts. Mol Cell.

[CR47] Kellum R, Alberts BM (1995). Heterochromatin protein 1 is required for correct chromosome segregation in Drosophila embryos. J Cell Sci.

[CR48] Kellum R, Raff JW, Alberts BM (1995). Heterochromatin protein 1 distribution during development and during the cell cycle in Drosophila embryos. J Cell Sci.

[CR49] Klattenhoff C, Xi H, Li C (2009). The drosophila HP1 homolog rhino is required for transposon silencing and piRNA production by dual-strand clusters. Cell.

[CR50] Kueng S, Oppikofer M, Gasser SM (2013). SIR proteins and the assembly of silent chromatin in budding yeast. Annu Rev Genet.

[CR51] Kumar J, Haldar S, Gupta N (2020). Swi6/HP1 binding to RNA-DNA hybrids initiates heterochromatin assembly at the centromeric dg-dh repeats in fission yeast. BioRxiv.

[CR52] Kwon SH, Florens L, Swanson SK (2010). Heterochromatin protein 1 (HP1) connects the FACT histone chaperone complex to the phosphorylated CTD of RNA polymerase II. Genes Dev.

[CR53] Lachner M, O’Carroll D, Rea S (2001). Methylation of histone H3 lysine 9 creates a binding site for HP1 proteins. Nature.

[CR54] Larson AG, Elnatan D, Keenen MM (2017). Liquid droplet formation by HP1α suggests a role for phase separation in heterochromatin. Nature.

[CR55] Lehming N, Le Saux A, Schüller J, Ptashne M (1998). Chromatin components as part of a putative transcriptional repressing complex. Proc Natl Acad Sci.

[CR56] LeRoy G, Weston JT, Zee BM (2009). Heterochromatin protein 1 is extensively decorated with histone code-like post-translational modifications. Mol Cell Proteomics.

[CR57] Li Y, Kirschmann DA, Wallrath LL (2002). Does heterochromatin protein 1 always follow code?. Proc Natl Acad Sci U S A.

[CR58] Li Y, Danzer JR, Alvarez P (2003). Effects of tethering HP1 to euchromatic regions of the Drosophila genome. Development.

[CR59] Lin C-H, Li B, Swanson S (2008). Heterochromatin protein 1a stimulates histone H3 lysine 36 demethylation by the Drosophila KDM4A demethylase. Mol Cell.

[CR60] Lin C-H, Paulson A, Abmayr SM, Workman JL (2012). HP1a targets the Drosophila KDM4A demethylase to a subset of heterochromatic genes to regulate H3K36me3 levels. PLoS One.

[CR61] Liu L-P, Ni J-Q, Shi Y-D (2005). Sex-specific role of Drosophila melanogaster HP1 in regulating chromatin structure and gene transcription. Nat Genet.

[CR62] Lomberk G, Bensi D, Fernandez-Zapico ME, Urrutia R (2006). Evidence for the existence of an HP1-mediated subcode within the histone code. Nat Cell Biol.

[CR63] Lorentz A, Ostermann K, Fleck O, Schmidt H (1994). Switching gene swi6, involved in repression of silent mating-type loci in fission yeast, encodes a homologue of chromatin-associated proteins from Drosophila and mammals. Gene.

[CR64] Lu BY, Emtage PC, Duyf BJ (2000). Heterochromatin protein 1 is required for the normal expression of two heterochromatin genes in Drosophila. Genetics.

[CR65] Maines JZ, Park JK, Williams M, McKearin DM (2007). Stonewalling Drosophila stem cell differentiation by epigenetic controls. Development.

[CR66] Maison C, Almouzni G (2004). HP1 and the dynamics of heterochromatin maintenance. Nat Rev Mol Cell Biol.

[CR67] Maison C, Bailly D, Peters AHFM (2002). Higher-order structure in pericentric heterochromatin involves a distinct pattern of histone modification and an RNA component. Nat Genet.

[CR68] Maison C, Bailly D, Roche D (2011). SUMOylation promotes de novo targeting of HP1α to pericentric heterochromatin. Nat Genet.

[CR69] Meehan RR, Kao C-F, Pennings S (2003). HP1 binding to native chromatin in vitro is determined by the hinge region and not by the chromodomain. EMBO J.

[CR70] Minc E, Allory Y, Worman HJ (1999). Localization and phosphorylation of HP1 proteins during the cell cycle in mammalian cells. Chromosoma.

[CR71] Motamedi MR, Hong E-JE, Li X (2008). HP1 proteins form distinct complexes and mediate heterochromatic gene silencing by nonoverlapping mechanisms. Mol Cell.

[CR72] Muchardt C, Guillemé M, Seeler J-S (2002). Coordinated methyl and RNA binding is required for heterochromatin localization of mammalian HP1α. EMBO Rep.

[CR73] Nakayama J, Rice JC, Strahl BD (2001). Role of histone H3 lysine 9 methylation in epigenetic control of heterochromatin assembly. Science.

[CR74] Nielsen PR, Nietlispach D, Mott HR (2002). Structure of the HP1 chromodomain bound to histone H3 methylated at lysine 9. Nature.

[CR75] Nishibuchi G, Nakayama J (2014). Biochemical and structural properties of heterochromatin protein 1: understanding its role in chromatin assembly. J Biochem.

[CR76] Nishibuchi G, Machida S, Osakabe A (2014). N-terminal phosphorylation of HP1α increases its nucleosome-binding specificity. Nucleic Acids Res.

[CR77] Perrini B, Piacentini L, Fanti L (2004). HP1 controls telomere capping, telomere elongation, and telomere silencing by two different mechanisms in Drosophila. Mol Cell.

[CR78] Piacentini L, Pimpinelli S (2010). Positive regulation of euchromatic gene expression by HP1. Fly (Austin).

[CR79] Piacentini L, Fanti L, Berloco M (2003). Heterochromatin protein 1 (HP1) is associated with induced gene expression in Drosophila euchromatin. J Cell Biol.

[CR80] Piacentini L, Fanti L, Negri R (2009). Heterochromatin protein 1 (HP1a) positively regulates euchromatic gene expression through RNA transcript association and interaction with hnRNPs in Drosophila. PLoS Genet.

[CR81] Pokholkova GV, Koryakov DE, Pindyurin AV (2015). Tethering of SUUR and HP1 proteins results in delayed replication of euchromatic regions in Drosophila melanogaster polytene chromosomes. Chromosoma.

[CR82] Roach RJ, Garavís M, González C (2020). Heterochromatin protein 1α interacts with parallel RNA and DNA G-quadruplexes. Nucleic Acids Res.

[CR83] Sales-Gil R, Vagnarelli P (2020) How HP1 post-translational modifications regulate heterochromatin formation and maintenance. Cells 9(6):1460. 10.3390/cells9061460 10.3390/cells9061460 PMC7349378 32545538

[CR84] Sanulli S, Trnka MJ, Dharmarajan V (2019). HP1 reshapes nucleosome core to promote phase separation of heterochromatin. Nature.

[CR85] Saunders WS, Chue C, Goebl M (1993). Molecular cloning of a human homologue of Drosophila heterochromatin protein HP1 using anti-centromere autoantibodies with anti-chromo specificity. J Cell Sci.

[CR86] Savitsky M, Kravchuk O, Melnikova L, Georgiev P (2002). Heterochromatin protein 1 is involved in control of telomere elongation in Drosophila melanogaster. Mol Cell Biol.

[CR87] Schoelz JM, Feng JX, Riddle NC (2020). The Drosophila HP1 family is associated with active gene expression across chromatin contexts. BioRxiv.

[CR88] Schotta G, Ebert A, Dorn R, Reuter G (2003). Position-effect variegation and the genetic dissection of chromatin regulation in Drosophila. Semin Cell Dev Biol.

[CR89] Schwaiger M, Kohler H, Oakeley EJ (2010). Heterochromatin protein 1 (HP1) modulates replication timing of the Drosophila genome. Genome Res.

[CR90] Seeler J-S, Marchio A, Sitterlin D (1998). Interaction of SP100 with HP1 proteins: a link between the promyelocytic leukemia-associated nuclear bodies and the chromatin compartment. Proc Natl Acad Sci.

[CR91] Seo S, Mathison A, Grzenda A (2018). Mechanisms underlying the regulation of HP1γ by the NGF-PKA signaling pathway. Sci Rep.

[CR92] Shimada A, Murakami Y (2010). Dynamic regulation of heterochromatin function via phosphorylation of HP1-family proteins. Epigenetics.

[CR93] Shimada A, Dohke K, Sadaie M (2009). Phosphorylation of Swi6/HP1 regulates transcriptional gene silencing at heterochromatin. Genes Dev.

[CR94] Singh PB, Miller JR, Pearce J (1991). A sequence motif found in a Drosophila heterochromatin protein is conserved in animals and plants. Nucleic Acids Res.

[CR95] Smothers JF, Henikoff S (2000). The HP1 chromo shadow domain binds a consensus peptide pentamer. Curr Biol.

[CR96] Smothers JF, Henikoff S (2001). The hinge and chromo shadow domain impart distinct targeting of HP1-like proteins. Mol Cell Biol.

[CR97] Snowden AW, Gregory PD, Case CC, Pabo CO (2002). Gene-specific targeting of H3K9 methylation is sufficient for initiating repression in vivo. Curr Biol.

[CR98] Stewart MD, Li J, Wong J (2005). Relationship between histone H3 lysine 9 methylation, transcription repression, and heterochromatin protein 1 recruitment. Mol Cell Biol.

[CR99] Strom AR, Emelyanov AV, Mir M (2017). Phase separation drives heterochromatin domain formation. Nature.

[CR100] Tatarakis A, Behrouzi R, Moazed D (2017). Evolving models of heterochromatin: from foci to liquid droplets. Mol Cell.

[CR101] Trewick SC, Minc E, Antonelli R (2008). The JmjC domain protein Epe1 prevents unregulated assembly and disassembly of heterochromatin. EMBO J.

[CR102] van der Vlag J, den Blaauwen JL, Sewalt RGAB (2000). Transcriptional repression mediated by polycomb group proteins and other chromatin-associated repressors is selectively blocked by insulators. J Biol Chem.

[CR103] Wang G, Ma A, Chow CM (2000). Conservation of heterochromatin protein 1 function. Mol Cell Biol.

[CR104] Wang X, Pan L, Wang S (2011). Histone H3K9 trimethylase eggless controls germline stem cell maintenance and differentiation. PLOS Genet.

[CR105] Wiese M, Bannister AJ, Basu S (2019). Citrullination of HP1γ chromodomain affects association with chromatin. Epigenetics Chromatin.

[CR106] Yamaguchi K, Hidema S, Mizuno S (1998). Chicken chromobox proteins: cDNA cloning of CHCB1, -2, -3 and their relation toW-heterochromatin. Exp Cell Res.

[CR107] Ye Q, Worman HJ (1996). Interaction between an integral protein of the nuclear envelope inner membrane and human chromodomain proteins homologous to Drosophila HP1. J Biol Chem.

[CR108] Yin H, Sweeney S, Raha D (2011). A high-resolution whole-genome map of key chromatin modifications in the adult Drosophila melanogaster. PLoS Genet.

[CR109] Zeng A, Li Y-Q, Wang C (2013). Heterochromatin protein 1 promotes self-renewal and triggers regenerative proliferation in adult stem cells. J Cell Biol.

[CR110] Zhao T, Eissenberg JC (1999). Phosphorylation of heterochromatin protein 1 by casein kinase II is required for efficient heterochromatin binding in Drosophila. J Biol Chem.

[CR111] Zhao T, Heyduk T, Eissenberg JC (2001). Phosphorylation site mutations in heterochromatin protein 1 (HP1) reduce or eliminate silencing activity. J Biol Chem.

[CR112] Zofall M, Grewal SIS (2006). Swi6/HP1 recruits a JmjC domain protein to facilitate transcription of heterochromatic repeats. Mol Cell.

